# ERS/ESTS/ESTRO/ESR/ESTI/EFOMP statement on management of incidental findings from low dose CT screening for lung cancer

**DOI:** 10.1093/ejcts/ezad302

**Published:** 2023-10-07

**Authors:** Emma L O'Dowd, Ilona Tietzova, Emily Bartlett, Anand Devaraj, Jürgen Biederer, Marco Brambilla, Alessandro Brunelli, Joanna Chorostowska, Herbert Decaluwe, Dirk Deruysscher, Walter De Wever, Matthew Donoghue, Aurelie Fabre, Mina Gaga, Wouter van Geffen, Georgia Hardavella, Hans-Ulrich Kauczor, Anna Kerpel-Fronius, Jan van Meerbeeck, Blin Nagavci, Ursula Nestle, Nuria Novoa, Helmut Prosch, Mathias Prokop, Paul Martin Putora, Janette Rawlinson, Marie-Pierre Revel, Annemiek Snoeckx, Giulia Veronesi, Rozemarijn Vliegenthart, Sabine Weckbach, Torsten G Blum, David R Baldwin

**Affiliations:** Nottingham University Hospitals NHS Trust, Nottingham, UK; University of Nottingham, Faculty of Medicine and Health Sciences, Nottingham, UK; Charles University, First Faculty of Medicine, Department of Tuberculosis and Respiratory Diseases, Prague, Czech Republic; Royal Brompton and Harefield NHS Foundation Trust, Radiology, London, UK; Royal Brompton and Harefield NHS Foundation Trust, Radiology, London, UK; University of Heidelberg, Diagnostic and Interventional Radiology, Heidelberg, Germany; German Center for Lung Research DZL, Translational Lung Research Center TLRC, Heidelberg, Germany; University of Latvia, Faculty of Medicine, Riga, Latvia; Christian-Albrechts-Universität zu Kiel, Faculty of Medicine, Kiel, Germany; Azienda Ospedaliero-Universitaria Maggiore della Carità di Novara, Novara, Italy; St. James's University Hospital, Department of Thoracic Surgery, Leeds, UK; Institute of Tuberculosis and Lung Diseases, Warsaw, Genetics and Clinical Immunology, Warsaw, Poland; University Hospitals Leuven, Thoracic Surgery, Leuven, Belgium; Maastricht University Medical Centre, Department of Radiation Oncology (MAASTRO Clinic), GROW-School for Oncology and Developmental Biology, Limburg, The Netherlands; Universitaire Ziekenhuizen Leuven, Radiology, Leuven, Belgium; Galway Clinic Doughiska, Galway, Ireland; University College Dublin School of Medicine, Histopathology, Dublin, Ireland; Sotiria General Hospital of Chest Diseases of Athens, 7th Respiratory Medicine Department, Athens, Greece; Medical Centre Leeuwarden, Department of Respiratory Medicine, Leeuwarden, The Netherlands; University of Groningen, University Medical Center Groningen, Department of Pulmonary Diseases, Groningen, The Netherlands; Sotiria General Hospital of Chest Diseases of Athens, Respiratory Medicine, Athens, Greece; University of Heidelberg, Diagnostic and Interventional Radiology, Heidelberg, Germany; German Center for Lung Research DZL, Translational Lung Research Center TLRC, Heidelberg, Germany; National Koranyi Institute of Pulmonology, Department of Radiology, Budapest, Hungary; UZ Antwerpen, MOCA, Edegem, Belgium; Institute for Evidence in Medicine, Medical Center – University of Freiburg, Faculty of Medicine, University of Freiburg, Freiburg im Breisgau, Germany; Kliniken Maria Hilf GmbH Monchengladbach, Nordrhein-Westfalen, Germany; University Hospital of Salamanca, Thoracic Surgery, Salamanca, Spain; Medical University of Vienna, Department of Biomedical Imaging and Image-guided Therapy, Vienna, Austria; Radboud University Nijmegen Medical Center, Department of Radiology, Nijmegen, The Netherlands; Kantonsspital Sankt Gallen, Radiation Oncology, Sankt Gallen, Switzerland; Inselspital Universitatsspital Bern, Radiation Oncology, Bern, Switzerland; European Lung Foundation, Tipton, UK; Cochin Hospital, APHP, Radiology Department, Paris, France; Université de Paris, Paris, France; University Hospital Antwerp, Radiology, Edegem, Belgium; Humanitas Research Hospital, Division of Thoracic and General Surgery, Rozzano, Italy; UMCG, Groningen, The Netherlands; UniversitatsKlinikum Heidelberg, Heidelberg, Germany; Bayer AG, Research and Development, Pharmaceuticals, Radiology, Berlin, Germany; HELIOS Klinikum Emil von Behring GmbH, Lungenklinik Heckeshorn, Berlin, Germany; University of Nottingham, Faculty of Medicine and Health Sciences, Nottingham, UK; Nottingham University Hospitals NHS Trust, Department of Respiratory Medicine, Nottingham, UK

## Abstract

**Background:**

Screening for lung cancer with low radiation dose computed tomography has a strong evidence base, is being introduced in several European countries and is recommended as a new targeted cancer screening programme. The imperative now is to ensure that implementation follows an evidence-based process that will ensure clinical and cost effectiveness. This European Respiratory Society (ERS) task force was formed to provide an expert consensus for the management of incidental findings which can be adapted and followed during implementation.

**Methods:**

A multi-European society collaborative group was convened. 23 topics were identified, primarily from an ERS statement on lung cancer screening, and a systematic review of the literature was conducted according to ERS standards. Initial review of abstracts was completed and full text was provided to members of the group for each topic. Sections were edited and the final document approved by all members and the ERS Science Council.

**Results:**

Nine topics considered most important and frequent were reviewed as standalone topics (interstitial lung abnormalities, emphysema, bronchiectasis, consolidation, coronary calcification, aortic valve disease, mediastinal mass, mediastinal lymph nodes and thyroid abnormalities). Other topics considered of lower importance or infrequent were grouped into generic categories, suitable for general statements.

**Conclusions:**

This European collaborative group has produced an incidental findings statement that can be followed during lung cancer screening. It will ensure that an evidence-based approach is used for reporting and managing incidental findings, which will mean that harms are minimised and any programme is as cost-effective as possible.

## Teaser

Lung cancer screening with low radiation dose computed tomography commonly detects incidental findings. This collaborative statement reviews the evidence and how this informs a clinically effective approach to these findings. https://bit.ly/3qlrlD3

## INTRODUCTION AND SCOPE

Low dose computed tomography (LDCT) screening for lung cancer is being implemented in some European countries, while others are still evaluating the health economics [1]. One of the areas of uncertainty stems from the fact that, unlike the other cancer screening programmes currently in progress, LDCT provides much more information than required for lung cancer detection. Incidental findings are detected frequently and have the potential to benefit or harm the participant, and their management adds costs [2–4]. Thus, included in many trial protocols and implemented programmes are guidelines on the management of incidental findings [5, 6]. These set out the possible findings and recommend actions, which may include referral for work-up, additional imaging or investigation, correlation with clinical features and context, or avoidance of any action. However, it is unclear to what extent active screening sites mandate and use protocols for incidental findings and how these protocols differ [7]. The aim of this task force was to provide a European clinical practice statement on the management of incidental findings encountered during computed tomography (CT) screening for lung cancer. The steps to achieving this aim were:

1. to define the incidental findings that are commonly encountered on LDCT screening;
2. to establish which findings are common enough to require statements on their management;
3. to review any existing guidelines or statements for each common incidental finding;
4. to recommend new guideline developments where needed;
5. to suggest a standard reporting format for management of incidental findings;
6. to recommend pathways for the further management of incidental findings as far as needed;
7. to discuss variations and their underlying rationale in recommendations that may apply in different countries, taking into account varying cost-effectiveness considerations;
8. to produce a series of statements on the reporting of individual incidental findings based on current guidelines/recommendations that can be used in the implementation of LDCT screening.

### Legal statement/remit

LDCT is not suitable to look for extrapulmonary abnormalities, as the screening test is specifically to detect lung cancer and is not optimised for other diseases. Indeed, LDCT is suboptimal for diagnosis and evaluation of many soft tissue abnormalities. This document focuses on those incidental findings where there is evidence to support an impact or change to patient management and those that may be reliably detected using LDCT. These findings will be communicated to either the treating clinician and/or the participant. Participants should be consented appropriately to reflect this prior to participating in screening.

## METHODS

The assembly of the task force was coordinated by the European Respiratory Society (ERS) following approval by the ERS Management Group in March 2020. It is a collaborative venture between ERS, European Society of Thoracic Surgeons (ESTS), European Society for Radiation Oncology (ESTRO), European Society of Radiology (ESR), European Society of Thoracic Imaging (ESTI) and European Federation of Organisations for Medical Physics (EFOMP), each of which provided representatives and agreed a formal memorandum of understanding. The expertise in the group covered most of the core specialisms involved in lung cancer screening, including radiology, pulmonology, thoracic surgery, radiation oncology and nuclear medicine. The task force received support from ERS methodologists throughout the project. The task force was further enhanced by involvement of a patient representative from the European Lung Foundation. Six meetings were held (five virtual and one in person at the ERS International Congress in 2022). All members of the task force signed conflict of interest disclosures at the beginning of the project and updated them at project finalisation or when any new relevant conflict of interest appeared. Conflicts of interest were managed according to ERS policy.

The first exercise was to develop a list of topics for evaluation. A comprehensive list of incidental findings was discussed, derived primarily from a recent ERS publication, written by several members of this task force [8]. The list was circulated to all members, who were asked to score each topic according to whether the finding was: 1) common enough to be included; 2) had a potential to result in clinically important variation in radiology reporting; 3) could be a finding requiring urgent action or was clinically serious; and 4) whether the topic would be better in the generic section or whether a full evaluation of the literature was required. Each category was scored as “yes”, “no” or “unsure”. The voting was coordinated by D.R. Baldwin and was not blinded. Votes were received from 14 task force members (12 senior experts and two early/mid-career members). The results were then discussed in two online meetings, during which there was clear consensus by the 28 (of 32) expert members of the task force who attended the meeting. The final list was circulated, and the remaining members asked if they had any objections; none were received after a period of 1 month. Topics were divided into those considered to require detailed standalone evaluation and those suitable for a generic statement. Generic statements cover combinations of incidental findings and statements across multiple incidental findings. Thresholds for reporting of many incidental findings are not clearly defined and may depend on the radiologist or the healthcare team.

Systematic literature searches were performed in MEDLINE and Cochrane Library in December 2021, covering a period from 2010 to 2021 (search terms shown in appendix A). In addition, task force members were asked to source government and other institutional documents that might be of relevance. Titles and abstracts were screened by two reviewers (I. Tietzova and D.R. Baldwin) independently using Covidence (www.covidence.org). Discordant abstracts were arbitrated by a third reviewer (T.G. Blum). Full text screening of records was conducted by two or more task force members independently and reference lists of all included records were examined for additional relevant citations up to July 2022. Only studies written in English, or for which an English translation was available, were included. The screening results are presented in appendix B using the Preferred Reporting Items for Systematic Reviews and Meta-Analyses (PRISMA) flow diagram [9].

The evidence reviewed for this statement was restricted to that drawn from lung cancer CT screening trials and programmes for all topics, unless stated otherwise in the relevant section. Included articles were classified according to which incidental finding they included (they could have multiple classifications). Some articles potentially covered the topic of incidental findings in general and were given a general classification for review for all topics.

## RESULTS

### Topics

Table 1 shows the list of incidental findings that became the subject of topics (see above for methodology). Based on expert consensus, nine incidental findings were selected for a specific review and section, eight of which were intrathoracic.

**Table 1:** Incidental findings considered by the task force

Finding	Topic review category^#^
Interstitial lung abnormalities	Specific
Emphysema	Specific
Bronchiectasis	Specific
Consolidation	Specific
Coronary artery calcification	Specific
Aortic valve disease	Specific
Mediastinal mass	Specific
Mediastinal lymph nodes	Specific
Thyroid abnormalities	Specific
Bronchial wall thickening	Generic
Respiratory bronchiolitis-associated interstitial lung disease	Generic
Pleural effusions and pleural thickening	Generic
Pneumothorax and pneumomediastinum	Generic
Diaphragm abnormalities	Generic
Tuberculosis	Generic
Cardiac decompensation/pericardial effusion	Generic
Aortic aneurysms	Generic
Breast nodules	Generic
Liver lesions	Generic
Renal lesions	Generic
Bone abnormalities	Generic
Adrenal lesions	Generic

# : “specific” refers to topics considered most important and frequent, which were reviewed as standalone topics, and “generic” to topics of lower importance or infrequent that were grouped into a generic category, suitable for a general statement.

### Summary of statements

#### Interstitial lung abnormality

- Most studies report a prevalence of incidentally detected interstitial lung abnormality (ILA) in CT screening for lung cancer to be between 3% and 10%, associated with age and smoking.
- In the context of CT screening for lung cancer, ILAs are incidental findings of non-dependent lung abnormalities including reticular patterns, traction bronchiectasis, non-emphysematous cyst and ground glass opacities. ILA does not include smoking-related respiratory bronchiolitis.
- Quantification of ILA is recommended by some guidance, but it is unclear how this is best achieved.
- ILAs may be characterised as non-subpleural, subpleural non-fibrotic and subpleural fibrotic, because these have implications for prognosis.
- Fibrotic ILAs are associated with progression and increased mortality.
- Although guidance indicates that ILA involving more than 5% of either the whole lungs or a lung zone could be referred for consultation, further work is needed to understand the downstream benefits of this approach. One other option would be surveillance imaging. This could be done as part of the routine screening programme (also see suggested research).

#### Emphysema

- The prevalence of emphysema detected during LDCT screening for lung cancer is dependent on inclusion criteria, but can be >50%.
- Severity of emphysema can be classified radiologically and may be useful to predict outcomes such as hospital admission and mortality.
- A suitable qualitative classification is mild (<25%) moderate (25–50%) and severe (>50%).
- Emphysema can be used as a predictor of lung cancer risk and is used to stratify lung cancer risk in screening nodule management protocols.
- It is not clear how identification of emphysema on CT screening influences outcome, but it may be prudent to refer those with moderate to severe radiological emphysema for clinical assessment.

#### Bronchiectasis

- The prevalence of bronchiectasis in lung cancer screening is variable, and may be a result of diagnostic criteria (mostly undefined) and difference in populations.
- One service guidance standard defines moderate to severe bronchiectasis as when the airway diameter is two or more times that of the adjacent artery and recommends referral for this.
- Bronchiectasis is associated with an increased recall rate due to a greater number of pulmonary nodules, but there is no evidence for a greater risk of lung cancer.
- There is no evidence for the benefit to the participant of early detection of bronchiectasis.
- Given the limited or absent evidence, particularly in the population with severe disease, referral for clinical assessment may be appropriate, but criteria for referral may need to be better defined.

#### Pleural effusion/thickening/pleural plaques

- Although the prevalence of malignant pleural disease is low in people screened for lung cancer, suspicious appearances, including a new effusion, malignant appearing pleural thickening or mass, are best managed according to existing guidelines that recommend referral for further clinical assessment and work-up.
- In some countries the finding of pleural plaques results in compensation. In these circumstances, the finding of pleural plaques could be reported.

#### Pneumothorax and pneumomediastinum

- In the absence of specific evidence, pneumothorax and pneumomediastinum are best managed according to existing clinical guidelines and therefore these findings would form part of the other findings section of radiology reports.

#### Diaphragmatic abnormalities

- Diaphragmatic abnormalities in the setting of lung cancer screening are rare and the clinical impact of these findings is likely to be low.

#### Consolidation

- Consolidation may be classified radiologically as “likely inflammatory” or “possibly malignant”.
- Inflammatory-appearing consolidation is frequently self-limiting at short interval CT (*e.g.* 3 months). Persisting consolidation at short interval CT or consolidation at a single CT where the appearances favour malignancy should be referred for further investigation.

#### Coronary artery calcification

- Coronary artery calcification (CAC) is a common finding on CT in lung cancer screening.
- CAC confers an adverse prognosis, in particular for cardiovascular events and mortality.
- CAC may be scored in a variety of ways, but simple visual scoring is able to stratify risk of adverse outcome.
- Guidelines recommend reporting of CAC and considering referral and primary preventive measures.
- An alternative method is to assess risk independent of CAC score.

#### Aortic valve calcification

- Aortic valve calcification (AVC) is a frequent finding in lung cancer screening, but severe AVC is uncommon.
- AVC severity is readily assessed by visual scoring, which is correlated with severity of aortic stenosis and outcomes.
- Guidelines and statements recommend that moderate/severe AVC is reported with referral recommended to primary care.

#### Other cardiac findings

- Thoracic aortic calcification (TAC) is a frequent finding in lung cancer screening and is associated with adverse outcomes.
- Guidelines do not recommend clinical assessment of TAC.
- Referral of participants with significantly dilated ascending thoracic aorta (≥40 or 42 mm) is recommended in guidelines.
- Recent evidence suggests a better threshold of ≥45 mm and cost-effectiveness of follow-up ≥50 mm.

#### Mediastinal lesions

- Anterior mediastinal masses may be stratified according to their size, position and density/texture.
- Higher risk anterior mediastinal masses are best investigated with contrast-enhanced magnetic resonance imaging (MRI) or CT.
- Although oesophageal malignancies are uncommon, benign pathology may be clinically relevant.
- Mediastinal and hilar lymphadenopathy >15 mm on the short axis that is unexplained may require further investigation and work-up, or at least short interval scanning (3 to 6 months). Morphological assessment of lymph nodes may also be useful. A threshold <15 mm will lead to many unnecessary referrals in the context of screening.

#### Thyroid lesions

- Thyroid abnormalities are seen on <5% of screening CT scans.
- Most of these are benign or indolent.
- Guidelines/consensus statements recommend referral for nodules ≥15 mm or those with suspicious features, such as local lymphadenopathy or punctate calcification.
- Evidence has suggested a 20 mm nodule size cut-off for referral may achieve a better balance of avoiding unnecessary work-up of benign nodules.

#### Breast lesions

- The rate of breast cancer seen in lung cancer screening is variable and very low in some studies.
- Most detected breast lesions are benign.
- Guidelines recommend referral of any breast lesion that was not previously known, or lesions that are not clearly cystic.

#### Adrenal lesions

- In the context of lung cancer screening, most incidental adrenal lesions up to 40 mm in size are benign.
- Guidelines state that lesions 10–40 mm or with attenuation >10 HU can be followed up at the next annual screening round or referred for further evaluation with contrast-enhanced CT or MRI. Adrenal lesions stable on CT over 12 months also may not require further investigation.
- Lesions <10 mm or <10 HU in density do not require further investigation.

A summary of the research questions and recommendations appears in appendix C.

## PULMONARY FINDINGS

### Interstitial lung abnormality

In a recent position paper authored by a Fleischner Society expert panel, ILA is defined as “specific findings on CT involving at least 5% of a lung zone that are potentially compatible with interstitial lung disease, in patients without clinical suspicion of the disease” [10]. The statement summarises the findings of the panel as follows: early ILAs are a common finding on CT, more so in older patients; they are an independent predictor of mortality; 20% progress over 2 years and 40% over 5 years; and individuals with the fibrotic subtype are most likely to progress. Specific management recommendations are made. The statement draws much evidence from CT screening for lung cancer as well as from other cohorts, such as occupational health screening.

#### Evidence review

109 full papers were reviewed from the total of 1650 abstracts reviewed plus an additional 12 papers were identified following full text review. Of these, seven papers contained details of ILA incidentally detected in the context of screening [11–17]. Two further papers were identified in screening cohorts, but not specifically lung cancer screening [18, 19]. In addition, there were three review papers [10, 20, 21], one health service document from the English National Health Service (NHS) [6] and two statements from the American College of Radiology (ACR) [5, 22], one of which applied to all incidental findings.

#### Prevalence

The prevalence of incidentally detected ILA varied between 1.2% and 16.7% [11, 14, 18, 19]. In the Multicentric Italian Lung cancer Detection (MILD) trial, 692 current or former smokers (having quit smoking ≤10% years before recruitment) with ≥10 pack-years smoking history, age ≥49 years and no history of cancer within the previous 5 years, had a crude prevalence of ILA of 3.6% (28 participants, 95% CI 2.1–5.9%) [16]. This increased to 10.1% (95% CI 4.8–21.1%) after adjustment for age, sex and smoking status. Only two participants had a usual interstitial pneumonia (UIP) pattern. An analysis of 884 participants at a single site from the National Lung Screening Trial (NLST) found a prevalence of 9.7% [13]. In 951 participants in a CT screening programme between 2010 and 2014, “ILD” was seen in 6.6% [15]. Significant univariate predictors of interstitial lung disease (ILD) were male sex (p=0.003), older age (p<0.0001), higher number of pack-years of cigarette smoking (p=0.0003) and greater severity of emphysema (p=0.004), but only age and male sex remained significant in the multivariate analysis.

#### Types of ILA found in LDCT screening

Types of ILA identified in NLST included non-fibrotic (ground glass opacities, mosaic attenuation, consolidation) and fibrotic ILA (ground glass opacities with reticular abnormality, reticular abnormality, honeycombing) [13]. In the MILD trial, a UIP-like pattern, other chronic interstitial pneumonia-like pattern, respiratory bronchiolitis-like pattern, and intermediate pattern were described [16]. The respiratory bronchiolitis pattern was most common. In reports from the International Early Lung Cancer Action Project (IELCAP), the most common pattern of ILD (ILA was not reported) was peripheral fibrosis without honeycombing involving multiple lobes [15].

#### Prognosis

Four papers gave information about the progression of incidentally detected ILA [11–13, 15]. Progression was shown in 20–60% of cases. Fibrotic pattern ILA was related to progression. A study using data from NLST showed 49% of non-fibrotic ILA improved while 37% of fibrotic ILA worsened. In an analysis of the Danish Lung Cancer Screening Trial (DLCST), participants with ILA were more likely to be diagnosed with ILD (HR 4.9, 95% CI 1.8–13.3; p=0.008), COPD (HR 1.7, 95% CI 1.2–2.3; p=0.01), pneumonia (HR 2.0, 95% CI 1.4–2.7; p<0.001), lung cancer (HR 2.7, 95% CI 1.8–4.0; p<0.001) and respiratory failure (HR 1.8, 95% CI 1.1–3.0; p=0.03). In IELCAP, honeycombing was associated with progression and extent of fibrosis. The proportion of ILAs subsequently diagnosed as ILD and treated was investigated in a study of 1853 participants undergoing LDCT screening [17]. Of these participants 78 (4.2%) had ILA extent of >5% and 43 (2.3%) underwent ILD assessment. Cough and/or dyspnoea was reported by 23 out of 43 (53.5%) participants and, after clinical work-up, ILD was diagnosed in 28 (1.5% of total). A total of 11 patients were treated.

#### Statements and health service documents

The ACR recommendation for managing incidental lung findings in thoracic CT considers ILA in the context of subpleural, predominantly basal reticular opacities and recommends referral for a pulmonary consultation where traction bronchiectasis is seen with or without honeycombing. If there are additional findings, such as diffuse nodules, ground glass opacity or cysts, then thin section CT with expiratory and prone imaging is recommended [5]. This is only recommended for abnormalities affecting more than 5% of the lungs. The ACR Lung Cancer Screening CT Incidental Findings Quick Reference Guide (QRG) recommends a pulmonary consultation for ILD and does not refer to ILA.

The NHS England Quality Assurance Standard (NHSE QAS) for the Targeted Lung Health Check Programme (TLHC) [6] makes specific management recommendations for ILA. All ILA >5% should be reported, but referral for pulmonology consultation should only occur if there is more than 10% reticulation of the lungs. In cases of 5–10% affected lung, spirometry should be performed for correlation. Referral can be considered if spirometry is abnormal. Less than 5% ILA does not require recording or action. The document states that there should be an option for review at a screening review meeting. Only significant CT results should be communicated to the participant and the general practitioner.

Accepting that the Fleischner Society position paper included all incidentally detected ILA, the recommendations are similar to ACR and NHS [10]. However, they noted some bias towards populations of people who smoke or have smoked (actually relevant to screening populations) and a need to do more research on histopathological correlation. To facilitate this, it is recommended that all pathologists record details of non-neoplastic lung parenchymal changes. The paper recommends that there be clarification of the criteria for quantification and the role of quantitative measurements (baseline and interval changes). It is recommended that reporting includes axial and craniocaudal distribution, the presence of ground glass abnormality, reticular abnormality, traction bronchiectasis, honeycombing and cysts. ILA should be categorised into non-subpleural, subpleural non-fibrotic or subpleural fibrotic. In terms of clinical evaluation, it is recommended that ILA be distinguished from ILD, risk factors for progression identified and follow-up planned.

#### Summary

ILAs are a common finding in CT screening for lung cancer. In this context they are independently associated with increased mortality but only some progress after a 2-year interval, the maximum interval likely between screens. They are more common in older individuals, those who smoke tobacco and in those with COPD. The fibrotic subtype may be more likely to progress to ILD. Only a small fraction of those with ILA identified on screening will have an ILD and require treatment. Monitoring of progress of ILA can be achieved during routine CT screening rounds without need for intervention in those who do not progress and in those with limited disease. Radiological reporting of ILAs should reflect this.

#### Statements

- Most studies report a prevalence of incidentally detected ILA in CT screening for lung cancer to be between 3% and 10%, associated with age and smoking [11, 14, 18, 19].
- In the context of CT screening for lung cancer, ILAs are incidental findings of non-dependent lung abnormalities including reticular patterns, traction bronchiectasis, non-emphysematous cyst and ground glass opacities [13, 15, 16]. ILA does not include smoking-related respiratory bronchiolitis.
- Quantification of ILA is recommended by some guidance [5, 6], but it is unclear how this is best achieved.
- ILAs may be characterised as non-subpleural, subpleural non-fibrotic and subpleural fibrotic, because these have implications for prognosis [11–13, 15].
- Fibrotic ILAs are associated with progression and increased mortality [11–13, 15].
- Although guidance indicates that ILA involving more than 5% of either the whole lungs or a lung zone could be referred for consultation, further work is needed to understand the downstream benefits of this approach. One other option would be surveillance imaging. This could be done as part of the routine screening programme (also see suggested research).

#### Suggested research

- How should ILA be quantified?
- What is the role of artificial intelligence in the classification and quantification of ILA?
- How should ILA and ILD be consistently differentiated?
- How should you follow-up ILA and does it affect prognosis?
- Is the identification of ILA an opportunity for therapeutic prevention of progression, and does this differ according to subtype of ILA on CT?
- What are the thresholds for further action (*i.e.* clinical assessment) beyond observation along with the routine screening intervals?

#### Emphysema

Emphysema is defined as the permanent enlargement of airspaces distal to the terminal bronchiole with destruction of alveolar walls [23]. On CT imaging, emphysema can be assessed qualitatively or quantitatively, the latter by assessing lung density (Hounsfield units; HU) below a defined CT threshold. This review summarises the implications of identification of emphysema on LDCT screening for lung cancer.

#### Evidence review

After title and abstract screening, 30 full papers were reviewed and a further 14 identified following full text review. In addition, we identified one health service document (NHSE QAS) [6] and two statements (American Association of Thoracic Surgery, and European position statement on lung cancer screening) [24, 25].

#### Prevalence

The prevalence of emphysema detected by CT in lung screening cohorts was described in 22 papers (including cohort and case–control studies) [14, 26–46]. The prevalence of emphysema detected in lung screening cohorts varied between 2.9% and 57% [36, 46]. The wide variation in prevalence can be explained by differing inclusion criteria, CT scanning parameters, and methodology for assessing emphysema on CT (qualitative, semi-quantitative, quantitative).

#### Prognosis

Several studies have investigated the relationship between emphysema and lung cancer risk, and emphysema and lung cancer mortality. Most studies demonstrate that the presence of emphysema on CT, when visually assessed, is significantly associated with increased lung cancer risk [26, 30, 35, 37, 40, 47–49]. Emphysema phenotype may be important. In a study of predominantly male screening participants, Gonzalez *et al*. [48] reported that the centrilobular subtype of emphysema is associated with the highest risk of lung cancer after adjusting for confounders (OR 34.3, 95% CI 25.5–93.3). In contrast, a small number of studies quantitatively assessing emphysema severity using automated methods have either failed to demonstrate a relationship between the severity of emphysema and lung cancer risk [39, 50–52], or demonstrated only a weak relationship [27].

In a cohort of 9047 asymptomatic screening patients, Zulueta *et al.* [41] reported that the presence of visually determined emphysema was a significant predictor of death from lung cancer (HR 1.7, 95% CI 1.1–2.5; p = 0.013), after adjusting for confounders. However, the same study demonstrated that when assessing emphysema extent, only marked emphysema was an independent risk factor for death from lung cancer. Gallardo-Estrella *et al.* [53] found that a quantified normalised emphysema score (adjusting for variations in emphysema scores caused by differences in slice thickness, reconstruction kernel and image noise) is a predictor of both all-cause mortality and lung cancer mortality. Sverzellati *et al.* [54] reported that in the MILD trial, neither the extent of emphysema nor the mean lung attenuation was predictive of all-cause mortality (median follow-up 36 months).

In a retrospective study of 5590 screening patients at two screening centres, Gazourian *et al.* [55] investigated the relationship between the presence of emphysema (qualitatively assessed) and all-cause hospital admission, COPD-related hospital admission and pneumonia-related hospital admission. At one site, emphysema was categorised as absent, mild, moderate or marked; moderate or marked emphysema was associated with COPD-related hospital admission (HR 1.64, 95% CI 1.14–2.36; p = 0.007) and all-cause hospital admission (HR 1.15, 95% CI 1.03–1.31; p = 0.014). At the second site, emphysema was categorised as present or absent, and the presence of emphysema was associated only with COPD-related admission (HR 2.05, 95% CI 1.07–3.95; p = 0.031).

#### Detection of undiagnosed COPD

National and international guidelines (not specific to lung cancer screening) do not currently recommend population-wide screening for COPD in the absence of symptoms [56–58]. Several papers describe the relationship between CT-detected emphysema and airflow obstruction, without direct correlation with participant symptoms, or in asymptomatic participants [26, 29, 59–62]. Two studies describe the inter-relationship between CT emphysema, lung function and symptoms in screening cohorts [33, 45]. In the Pittsburgh Lung Screening Study (PLuSS), 1340 out of 3183 (42.1%) screened participants had CT-detected emphysema, scored using a five-level semiquantitative scale [45]. Of these 1340, 866 (64.6%) had obstructive spirometry, but only 660 out of the 866 (76.2%) had symptoms. A further 474 out of 3183 (14.9%) participants had emphysema with no evidence of airway obstruction. 370 out of 3183 (11.6%) participants had airflow obstruction and symptoms (COPD) without evidence of emphysema on CT. The relationship between emphysema severity and lung function and symptoms was not described.

In the Lung Screen Uptake Trial, 560 out of 986 (57%) participants undergoing spirometry had airway obstruction, of whom only 309 of the 560 (55.2%) had emphysema on CT [33]. In this cohort of participants with airway obstruction and emphysema, 197 out of 309 (63.8%) were symptomatic. Furthermore, among 297 screened participants with no prior diagnosis of COPD, but airflow obstruction, 47 (15.8%) were symptomatic, but with no CT evidence of emphysema. The presence of symptoms was associated only with severe emphysema in this study (OR 4.00, 95% CI 1.57–10.2). The presence of airflow obstruction was a better predictor of patient symptoms than emphysema.

These studies demonstrate that the presence of emphysema on lung screening CT can be used to identify symptomatic undiagnosed COPD, but with limited overall sensitivity and positive predictive value. Only limited evidence exists to suggest that identification of undiagnosed COPD results in changes to patient management. In a cohort study of 55 lung cancer screening participants referred to primary care for COPD assessment on the basis of spirometry findings and symptoms, Bartlett *et al*. [4] found that only 16 (29.1%) participants received a new respiratory diagnosis, and changes to patient management (pharmacotherapy or pulmonary rehabilitation) occurred in only seven out of the 55 (12.7%) referred.

#### Emphysema identification to improve risk stratification for lung cancer screening

Sanchez-Salcedo *et al*. [34] investigated the benefits of identifying emphysema to improve risk stratification for ongoing annual lung cancer screening after a baseline screen. Applying NLST criteria for screening selection to Pamplona (P)-IELCAP and PLuSS screening cohorts (both with broader inclusion criteria for screening), they find that annual screening of those meeting NLST criteria and/or with emphysema on a baseline scan would have detected 88% and 95% of incident lung cancer in the P-IELCAP and PLuSS screening cohorts, but could reduce the number of participants requiring annual screening by 52%.

The presence of emphysema can also be used to determine lung cancer risk in participants with lung nodules. The Brock/PanCan model [63], which includes emphysema as a risk factor, was derived from data from 1871 individuals with 7008 lung nodules enrolled in the Pan-Canadian Early Detection of Lung Cancer (PanCan) study, and was initially validated in chemoprevention trials at the British Columbia Cancer Agency [63]. The Brock model is incorporated in several nodule management protocols, including British Thoracic Society nodule management guidelines [64], that have been modified for use in several screening trials and pilots in the UK [65–67].

#### Statements and health service documents

The NHSE QAS for the TLHC makes specific recommendations for action taken after the finding of emphysema [6]. The standard recommends the visual quantification of emphysema as mild (<25%), moderate (25–50%) or severe (>50%). The presence of emphysema should reinforce the need for participants to be offered smoking cessation where appropriate, and consideration can be given to referral of those with moderate–severe (>25% of lungs involved) emphysema for respiratory review. The standard specifically states that emphysema should not be used to diagnose COPD.

The American Association for Thoracic Surgery guidelines from 2012 state that emphysema should be assessed (but not how this should be done), without specific associated management recommendations [24].

The European position statement on lung cancer screening states that identification of emphysema may enhance the cost-effectiveness of screening [25].

#### Summary

Emphysema is commonly detected on LDCT screening studies. Emphysema (assessed visually, rather than quantitatively) is associated with increased lung cancer risk and lung cancer mortality. Further work is needed to understand how and whether this should impact on screening intervals. Emphysema on CT cannot be used to diagnose COPD but can be used to identify participants who may have undiagnosed symptomatic airflow obstruction. However, evidence is lacking as to whether this leads to improvement in outcomes or is cost-effective when incorporated into a lung cancer screening programme.

#### Statements

- The prevalence of emphysema detected during LDCT screening for lung cancer is dependent on inclusion criteria, but can be >50% [36, 46].
- Severity of emphysema can be classified radiologically and may be useful to predict outcomes such as hospital admission and mortality [53].
- A suitable qualitative classification is mild (<25%) moderate (25–50%) and severe (>50%).
- Emphysema can be used as a predictor of lung cancer risk and is used to stratify lung cancer risk in screening nodule management protocols [65–67].
- It is not clear how identification of emphysema on CT screening influences outcome, but it may be prudent to refer those with moderate to severe radiological emphysema for clinical assessment.

#### Suggested research

- What are the benefits of screening patients with emphysema (as a comorbidity) given the increased lung cancer mortality and all-cause mortality in this cohort?
- Given the relationship between emphysema and lung cancer risk, should emphysema (severity) on screening LDCT influence subsequent LDCT screening and its interval?
- Does the detection of emphysema and subsequent investigation of an individual for COPD lead to changes in patient management, behaviour (such as smoking cessation), treatment (including pharmacotherapy) and, most importantly, outcome in the screening context?
- What is the cost-effectiveness of COPD case-finding through reporting emphysema in the context of screening for lung cancer?
- How can the method for quantification of emphysema in LDCT be optimised, and is there additional value of emphysema quantification?

#### Bronchiectasis

Bronchiectasis is defined as irreversible localised or diffuse bronchial dilatation [23]. On CT imaging, airways are regarded as bronchiectatic if their internal diameter is larger than the accompanying adjacent pulmonary artery, if there is a lack of tapering of the bronchial lumen, or visibility of bronchi within 1 cm of the pleura [68].

#### Evidence review

11 full papers and one health service document (NHSE QAS) [6] were reviewed.

#### Prevalence

The prevalence of bronchiectasis in lung cancer screening studies ranged from 0.2% to 18.9% [2, 14, 69–75]. One study used the original definition for bronchiectasis [72]. Another study reported the severity and extent of bronchiectasis according to the Bhalla score (a score originally derived for patients with cystic fibrosis) [75, 76]. Most papers did not report the method for assessing bronchiectasis. Thus, the wide variability in the prevalence of bronchiectasis may relate to diagnostic criteria, or possibly to inclusion criteria for lung cancer screening or exposure to previous infection in differing patient populations.

#### Prognosis

354 screened participants with bronchiectasis (11.6%) were compared to 354 control participants without bronchiectasis in the IELCAP study [75]. Participants with bronchiectasis were more likely to have lung nodules on a baseline screen (53.4% *versus* 17.8%; p<0.001) and had a higher false-positive rate (26% *versus* 17%; p = 0.003) than those without bronchiectasis. This resulted in higher follow-up rates and more antibiotic treatment in the participants with bronchiectasis. At subsequent screening rounds, those with bronchiectasis were more likely to have new or growing nodules (33.7% *versus* 23.5%; p<0.001). At baseline, there was no significant difference between the groups in the proportion of patients requiring CT biopsy (0.85% *versus* 0.56%; p = 0.654). The study was unable to make any conclusions about any association of bronchiectasis with lung cancer because there were only five prevalent and two incident cancers in the bronchiectasis group and one prevalent and five incident cancers in the control group. No other studies have described the prognostic implications of detecting bronchiectasis in lung cancer screening.

#### The role of healthcare professionals in managing screening-detected bronchiectasis

In the ProActive Lung CAncer Detection (PALCAD) study, all patients with bronchiectasis were reviewed in primary care, with a subset of participants with respiratory incidental findings (including emphysema, bronchiectasis and lung fibrosis) subsequently referred to secondary care [69]. However, no details are provided about how these patients were subsequently managed in primary and/or secondary care, or about their outcome. One UK screening study protocol recommends that only participants with severe bronchiectasis, defined as internal bronchial lumen diameter more than three times the diameter of the accompanying artery, should be referred to primary care. A primary care clinical review is recommended, with a referral to secondary care only in symptomatic individuals [66]. The referral pathways and/or management of bronchiectasis detected in screening is otherwise not described.

#### Statements and health service documents

The NHSE QAS for the TLHC includes recommendations that those with moderate or severe bronchiectasis, defined as internal bronchial luminal diameter more than two times the adjacent artery, should be referred to a respiratory clinic (if symptomatic) or to primary care with a view to prophylactic management of infection if required [6]. The ACR QRG suggests primary care assessment and consideration of a pulmonary referral [5].

#### Summary

Bronchiectasis is often detected in lung cancer screening studies. Those with bronchiectasis may have an increased recall rate for further screening, and a higher false-positive rate, but no evidence suggests an increased risk of lung cancer. To date, there are very few data regarding the prognostic benefits of identification of bronchiectasis, or the costs and cost-effectiveness of referral and further investigation of this condition.

#### Statements

- The prevalence of bronchiectasis in lung cancer screening is variable, and may be a result of diagnostic criteria (mostly undefined) and difference in populations [2, 14, 69–75].
- One service guidance standard defines moderate to severe bronchiectasis as when the airway diameter is two or more times that of the adjacent artery and recommends referral for this [6].
- Bronchiectasis is associated with an increased recall rate due to a greater number of pulmonary nodules, but there is no evidence for a greater risk of lung cancer [75].
- There is no evidence for the benefit to the participant of early detection of bronchiectasis.
- Given the limited or absent evidence, particularly in the population with severe disease, referral for clinical assessment may be appropriate, but criteria for referral may need to be better defined.

#### Suggested research

- How should bronchiectasis severity and extent be quantified?
- What is the role of artificial intelligence in the classification and quantification of bronchiectasis?
- Does the detection and quantification of bronchiectasis during screening result in meaningful beneficial changes to patient management, treatment (including pharmacotherapy) or outcome?
- What is the cost-effectiveness of detecting bronchiectasis in a lung cancer screening programme?

### Pleural effusions/thickening/pleural plaques

#### Evidence review

24 full papers were reviewed, as well as one additional paper identified following full text review. Of these, six papers contained details on pleural related findings in the context of lung cancer screening. In addition, there were two review papers.

In a sample of 1929 participants in the NELSON trial, pleural calcification and pleural plaques were considered “not clinically relevant” [70], and were found in 51 (2.6%) and 66 (3.4%) participants respectively. Pleural fluid was categorised as “possibly clinically relevant”, but was not present in any participant. In their report of the Korean Lung Cancer Screening (K-LUCAS) pilot, Lee *et al.* [14] found pleural effusion in only two out of 256 (0.8%) participants. Swensen *et al.* [72] found a pleural effusion in four out of 1520 (0.3%) participants. The authors did not further address the possible importance of this finding.

A number of studies have focused on lung cancer screening in a population with asbestos contact. Pleural thickening in this population is common, with incidence ranging between 21.3% and 89.4% [77–79]. Pleural effusion, however, is rare (ranging from 0% to 2.1%), with a benign aetiology in most studies. Kato *et al.* [79] reported a pleural effusion in 45 out of 2132 (2.1%) participants, of whom six had lung cancer.

#### Statements and health service documents

The NHSE QAS recommends reporting size and laterality [6]. If malignant features are seen, participants should be referred for work-up or discussed at a screening review meeting.

The ACR QRG advises that when a new effusion, thickening or mass is seen on imaging, further evaluation by a primary care provider or pulmonary consultation should be considered [5].

#### Summary

The prevalence of pleural abnormalities in a general high risk population of lung cancer screening participants is low, with most being benign. In a screening population of participants with asbestos contact, pleural thickening and plaques are more common, but with a low risk of malignancy in the absence of suspicious pulmonary findings.

#### Statement

- Although the prevalence of malignant pleural disease is low in people screened for lung cancer, suspicious appearances, including a new effusion, malignant appearing pleural thickening or mass, are best managed according to existing guidelines that recommend referral for further clinical assessment and work-up.
- In some countries, the finding of pleural plaques results in compensation. In these circumstances, the finding of pleural plaques could be reported [80].

### Pneumothorax and pneumomediastinum

#### Evidence review

20 full papers and two reviews were reviewed.

No evidence was retrieved dealing with these findings within the context of LDCT screening. There is also no recommendation within the reviews and statements on incidental findings. Thus, it is assumed that these would be managed according to established clinical guidelines.

#### Statement

- In the absence of specific evidence, pneumothorax and pneumomediastinum are best managed according to existing clinical guidelines and therefore these findings would form part of the other findings section of radiology reports.

### Diaphragmatic lesions/abnormality

#### Evidence review

17 full papers were reviewed. Of these, only two papers contained details on diaphragmatic abnormalities.

A LDCT lung cancer screening study in 633 asbestos workers showed a diaphragm eventration in two participants (0.3%) [81]. A retrospective review of 320 LDCT participants reports five participants (1.6%) with a diaphragmatic hernia [2]. Neither study provides any additional information on clinical impact or relevance. Similarly, no specific recommendation is made in guidance documents and statements.

#### Summary

Diaphragmatic abnormalities in the setting of lung cancer screening are rare and the clinical impact of these findings is likely to be low [2, 81].

### Consolidation

#### Evidence review

In total, 19 full text papers were screened for information on management of consolidation in the context of lung cancer screening. In one screening study of 3800 patients, consolidation was found in 8.7% [82]. The opacities frequently resolved at follow-up imaging (79.8%) and lung cancer was identified in two patients. The authors from this study proposed that employing a protocol of repeat CT in 3 months for participants with consolidation would avoid unnecessary investigation in a substantial proportion of cases of self-limiting inflammatory opacities.

The NHSE QAS recognises the importance of morphological assessment by the radiologist in distinguishing consolidation that is more likely malignant from that more likely to be inflammatory. It recommends 3-month interval CT for consolidation that is more likely inflammatory, with referral for further investigation where malignancy is felt more likely [6]. It also states that “minor consolidation” is unlikely to be clinically significant and may not require reporting or follow-up. The ACR QRG makes no reference to consolidation [5] and the ACR pulmonary findings white paper simply describes the importance of differentiating consolidation from ground glass opacity [22].

#### Statements

- Consolidation may be classified radiologically as “likely inflammatory” or “possibly malignant” [6].
- Inflammatory-appearing consolidation is frequently self-limiting at short interval CT (*e.g.* 3 months). Persisting consolidation at short interval CT or consolidation at a single CT where the appearances favour malignancy should be referred for further investigation.

#### Suggested research

- What are the outcomes of protocols that manage screen-detected consolidation?
- Can artificial intelligence be used to better categorise consolidation at a single time-point CT?

## NON-PULMONARY FINDINGS

### Coronary artery calcification

The amount of CAC derived from ECG-synchronised, non-contrast cardiac CT is a strong, independent predictor of cardiovascular events [83]. Traditionally, quantification of CAC has been performed according to Agatston's method [84]. CAC scoring improves risk stratification of asymptomatic individuals, particularly those at intermediate risk based on cardiovascular risk factors, and is recommended in guidelines as a tool to determine whether preventive medication is indicated [83]. Compared to ECG-synchronised CT, (low dose) chest CT underestimates the presence and severity of CAC, but overall, there is high agreement in CAC score-based risk categorisation [85].

#### Evidence review

41 full papers were reviewed from the total of 1650 abstracts reviewed, as well as an additional paper identified following full text review. Of these, 22 papers contained details of CAC that was either incidentally detected or systematically investigated, in the context of lung cancer screening, and were included in evidence synthesis. The ACR QRG and the NHSE QAS both have recommendations on CAC reporting and management [5, 6]. The US Societies of Thoracic Radiology and of Cardiovascular Computed Tomography published guidelines on non-contrast, non-cardiac CT in 2016 [83] and, more recently, the British Societies of Cardiac Imaging, Cardiac CT and Thoracic Imaging produced a consensus statement on the evaluation and management of CAC on chest CT, including lung cancer screening, with a detailed discussion of evidence, scan protocol, CAC score methodologies and recommendations for reporting [86].

The 22 papers all concern lung cancer screening (sub)cohorts (n = 225–12 332), and nearly all report the prevalence and/or distribution of CAC severity. 11 studies applied the Agatston score to quantify CAC [54, 87–96], and another three studies used CAC volume [97–99]. In four studies, visual scoring (0–3: none, mild, moderate, severe) was used [55, 87, 100, 101], while five studies applied vessel-based, ordinal scoring (0–12: none, mild, moderate, severe for the four coronary arteries individually) [3, 87, 102–104]. Chiles *et al.* [87] compared three scoring systems (Agatston scoring, visual scoring, adapted ordinal scoring), and found good to high inter-reader agreement, and good agreement between visual scoring and CAC score categories, suggesting that simple visual scoring of CAC may be sufficient in lung cancer screening.

#### Prevalence

The prevalence of CAC ranged from 21% to 94%, with 13 of 18 studies reporting a prevalence well above 50% [3, 14, 54, 55, 87–94, 96, 100–104]. Overall, with rising age the presence of CAC increased, with prevalence in men higher than in women at any age [3, 92]. Results from NLST show that CAC is present in 72% of men aged 55–59 years *versus* 48% of women (median volume 39 *versus* 2 mm^3^), increasing to 96% of men aged 70–74 years *versus* 86% of women (median volume 367 *versus* 91 mm^3^) [92]. In a screening cohort based on National Comprehensive Cancer Network inclusion criteria (n = 4673), mild CAC was found in 28%, moderate CAC in 24% and severe CAC in 26% [55]. In a NELSON subcohort (n = 3111), Agatston score of 1–10 was found in 9.3%, 11–100 in 21%, 101–400 in 20% and >400 in 29% [96].

#### Prognosis

In total, 14 articles addressed correlation of CAC to outcomes after follow-up periods ranging from 1.8 to 9 years [54, 55, 87, 88, 90, 92, 93, 95–99, 102, 104]. Of these, two populations were each reported by two papers with the same follow-up period, but with different ways of measuring or categorising CAC severity [90, 91, 96, 99]. Five studies (including two with the same cohort and follow-up) used a case–control/cohort approach [87, 90–92, 102]. Except for one study, which reported all-cause hospitalisation [55], all studies used all-cause mortality and/or cardiovascular/coronary heart disease mortality or cardiovascular events as outcome(s). All studies found that the amount of CAC was independently associated with worse outcomes. For instance, in a NLST case–cohort analysis (n = 1442), compared to an Agatston score of 0, the multivariable-adjusted hazard ratio for coronary heart disease mortality was 1.27 (95% CI 0.69–2.53) for 1–100, 3.57 (95% CI 2.14–7.48) for 101–1000, and 6.63 (95% CI 3.57–14.97) for >1000 [87]. Corresponding hazard ratios for visual scoring categories were 2.09 (95% CI 1.30–4.16) for mild CAC, 3.86 (95% CI 2.02–8.20) for moderate CAC and 6.95 (95% CI 3.73–15.67) for severe CAC [87]. For Agatston score >400 compared to 0, the hazard ratios for cardiovascular mortality were 3.8 (95% CI 1.0–15) in DLCST (n = 1945) [93], and 2.62 (95% CI 1.90–3.68) in NLST (n = 12 332) [95], and hazard ratios for cardiovascular events were 2.87 (95% CI 1.13–7.27) in MILD (n = 1159) [54], and 12.58 (95% CI 5.42–29.16) in NELSON [96].

#### Statements and health service documents

The lung cancer screening-based management documents (by ACR and NHS) [5, 6] report that the presence of CAC should be noted, and overall visual scoring (none, mild, moderate, severe) performed (NHS document based on the most severely affected artery). The statements recommend primary care evaluation of cardiovascular risk factors in case of significant CAC.

#### Summary

CAC is a frequent finding in lung cancer screening, which is more common and severe in men and with increasing age. Major lung cancer screening trials have all investigated the prognostic value of CAC scoring (either by Agatston scoring or visual scoring) and have found strong associations between severity of CAC and worse outcomes. Current management documents all agree on the importance of noting and reporting the presence and severity of CAC on (low dose) chest CT, at least using a simple visual scoring (none, mild, moderate, severe). It has also been suggested that an alternative approach is to assess risk independent of CAC score and use the former as the sole method to determine the need for primary prevention [66].

#### Statements

- CAC is a common finding on CT in lung cancer screening [3, 14, 54, 55, 87–94, 96, 100–104].
- CAC confers an adverse prognosis, in particular for cardiovascular events and mortality [87, 93, 95, 96].
- CAC may be scored in a variety of ways, but simple visual scoring is able to stratify risk of adverse outcome [87].
- Guidelines recommend reporting of CAC and considering referral and primary preventive measures.
- An alternative method is to assess risk independent of CAC score [66].

#### Suggested research

- What is the impact on long term outcomes of primary prevention provoked by CAC scoring?
- How does risk stratification with CAC supplement existing risk stratification tools such as Q-Risk in individuals with a strong smoking history?
- Are quantitative methods of CAC evaluation better at predicting and improving outcomes?
- How far does appearance of (severe) CAC change the overall risk stratification, including eligibility for the screening programme?
- What is the role of artificial intelligence in the quantification of CAC and what is its impact on accuracy of prevention?
- What is the cost-effectiveness of reporting CAC?
- What is the impact on prognosis and management of the absence of coronary calcium on LDCT in lung screening populations?

#### Aortic valve calcification and other cardiovascular findings

In contrast to the extensive literature on coronary calcification in lung cancer screening, there is limited evidence on the presence and value of other cardiovascular findings on low dose chest CT, such as AVC. The evidence is reviewed below, and the existing management recommendations are reviewed.

#### Aortic valve calcification

AVC is often noted in patients undergoing ECG-synchronised CT imaging. The extent of AVC on CT is associated with the presence of clinically relevant aortic stenosis [86]; aortic valve Agatston score cut-offs for diagnosis of severe aortic stenosis are known. Comparison studies on aortic valve Agatston scoring results for ECG-synchronised CT and (low dose) chest CT are currently lacking.

#### Evidence review

Nine full papers were reviewed from the total of 1650 abstracts reviewed. Of these, four papers contained details of AVC, either incidentally detected or systematically investigated, in the context of lung cancer screening, and were included in evidence synthesis. The ACR QRG and the NHSE QAS both have recommendations on AVC reporting and management [5, 6].

The current evidence derives from five studies, including a NELSON sample, the Mount Sinai Early Lung Cancer Action Program (MS-ELCAP), two further lung cancer screening cohorts, and a self-referred cohort to a health promotion centre (62% (ex-)smokers), with cohort sizes ranging from 1225 to 8618 participants screened [99, 103–106]. The severity of AVC was assessed in different ways: two studies applied visual scoring of valve leaflet calcifications [103, 104] ranging from none (grade 0) to severe (grade 3), while four studies used quantification of AVC, either Agatston score [103, 105, 106] or calcification volume [99]. Strong correlation was found between categories based on visual scoring and based on Agatston scoring [103].

#### Prevalence

The prevalence of AVC ranged from 6.4% to 21%. Predictors of AVC were age, male sex, CAC and cardiovascular risk factors [103–106]. MS-ELCAP showed that 9.5% of participants had mild AVC, 2.1% moderate AVC, and 0.16% severe AVC [103]. Moderate/severe AVC had a sensitivity of 100% and specificity of 94% for moderate/severe aortic stenosis in those who underwent echocardiography. In the health promotion centre study, participants underwent echocardiography within 1 year; of those with AVC, 10% had aortic stenosis (of these, 23% had moderate/severe aortic stenosis). Median aortic valve Agatston score in people with aortic stenosis was 448 (172–1185) *versus* 48 (19–107) in those without; diagnostic accuracy was 0.92 [105]. A Polish screening study showed that 13.1% of all participants had any degree of AVC, but only 7.8% of these had calcium scores that were high enough to warrant echocardiography (an arbitrary calcium score cut-off of 900, using a modified Agatston method, was used by the authors as a threshold for further investigation). Of these who underwent echocardiography, 24% had severe, 37% moderate and 30% mild aortic stenosis [106].

#### Prognosis

In NELSON, during a median follow-up of 2.9 years, 6.0% of participants had a cardiovascular event [99]. AVC volume was significantly related to prognosis, with a multivariate-adjusted hazard ratio of 1.46 (95% CI 1.09–1.84) per 500 mm^3^ AVC volume. Median (interquartile range) AVC volume in those with an event was 1353 (302–3129) mm^3^ compared to 419 (74–1366) mm^3^ in those without [99]. Furthermore, in the study by Zhu *et al.* [104], with a median follow-up of 8.0 years, the hazard ratio of AVC presence was 1.39 (95% CI 1.01–1.93) for cardiovascular death. Combined with ordinal CAC scoring, the presence of AVC significantly increased event risk in those with more extensive CAC [104].

#### Statements and health service documents

The lung cancer screening-based management documents (by ACR and NHS) [5, 6] both recommend primary care evaluation for moderate/severe AVC. It recommends reporting moderate/severe AVC and, if clinically appropriate, a primary care referral for echocardiography.

#### Summary

AVC is visible in up to 20% of lung cancer screening CT scans. Either visual scoring or Agatston scoring can yield a measure of severity of AVC. The severity of AVC is related to presence of moderate/severe aortic stenosis and to cardiovascular event risk. According to current management documents, moderate/severe AVC should be reported on chest CT, including lung cancer screening scans.

#### Statements

- AVC is a frequent finding in lung cancer screening, but severe AVC is uncommon [103–106].
- AVC severity is readily assessed by visual scoring, which is correlated with severity of aortic stenosis and outcomes [104].
- Guidelines and statements recommend that moderate/severe AVC is reported with referral recommended to primary care.

#### Suggested research

- How does reporting of moderate/severe AVC in lung cancer screening impact on outcomes?
- What is the cost-effectiveness of reporting AVC?
- Do quantitative measurements improve identification of AVC that can be effectively treated, and does quantification lead to better outcomes?
- Can artificial intelligence be used to quantify AVC?

### Other cardiovascular findings

#### Evidence review

12 full papers were reviewed from the total of 1650 abstracts, plus an additional seven papers were identified following full text review. Of these, eight papers contained details of other cardiovascular findings, either incidentally detected or systematically investigated, in the context of lung cancer screening, and were included in evidence synthesis. The ACR QRG and the NHSE QAS both comment on other cardiovascular findings [5, 6].

The included articles all comprise lung cancer screening trials (CT-Risk, DLCST, MILD, MS-ELCAP NELSON, NLST). Most studies investigated calcification of the thoracic aorta (TAC) [89, 90, 97, 98, 107–109] or the mitral valve (MVC) [98, 99], or a combination [89], while two studies focused on vascular diameters [107, 108]. For the calcification reports, quantification included either Agatston scoring [89, 90, 109] or volume quantification [97–99]. Regarding vascular diameters, manual measurement of the cross-sectional diameter was performed [108], or automated derivation of the diameter based on cross-sectional area was used [107].

#### Prevalence

In the CT-Risk trial (n = 501 men with occupational exposure undergoing lung cancer screening; 72% current smokers), the prevalence of extracoronary calcifications (TAC, AVC, MVC) was 85%, with significant correlations to cardiovascular risk factors and risk scores [99]. In a NELSON case–control sample (n = 958), TAC was present in 97% [90]. In NLST (n = 5718; 62% men), TAC prevalence increased with age in both sexes [109]. In men, TAC prevalence ranged from 87.2% for 55–59 years to 99.5% for 70–74 years (median volume ranging from 153 to 1926 mm^3^). Corresponding percentages in women were 89.6% and 100% (median volume ranging from 188 to 2771 mm^3^). MVC is an infrequent finding. In a study aimed at deriving a prediction model for cardiovascular mortality, the median MVC volume in NLST (n = 23 096) and MILD (n = 2287) was 0 [98]. In a NELSON subcohort (n = 3111), the prevalence of MVC was 2.7% [99].

In the DLCST (n = 1987), the mean±sd ascending and descending aorta diameters were 35±4 mm and 27±2 mm, with a mean growth of 0.08–0.17 mm per year by comparing last and first screening CT scan [107]. In MS-ELCAP (n = 1949), the focus was on the main pulmonary artery (MPA) diameter and the ratio of MPA to the ascending aorta diameter [108]. Mean MPA diameter was 26.6±3.9 mm, and mean MPA/ascending aorta diameter ratio was 0.81. An abnormal MPA diameter (>34 mm) was found in 4.2%, and an abnormal ratio of MPA/ascending aorta diameter (>1.0) in 6.9% [108].

#### Prognosis

Three studies have investigated the prognostic value of TAC severity for (cardiovascular) mortality and cardiovascular events [90, 97, 109]. In 5718 NLST participants, during a median follow-up of 6.5 years, 30% died, including 7.7% due to cardiovascular disease. Compared to a score of 0–1000, men and women had increased risk of (cardiovascular) mortality in case of Agatston score of 1001–4000 and >4000, with multivariate-adjusted hazard ratio up to 2.47 (95% CI 1.75–3.49) in men and 1.85 (95% CI 1.05–3.26) in women. In a NELSON case–cohort study, median follow-up was 22 months [90]; increasing TAC in Agatston score quartiles resulted in significantly increased risk of events, with hazard ratios up to 5.45 (95% CI 1.73–17.16) for all-cause mortality and 2.25 (95% CI 1.17–4.34) for cardiovascular events. In another NELSON sample, Mets *et al.* [97] reported a hazard ratio for cardiovascular events of 1.10 (95% CI 1.03–1.18) per 500 mm^3^ TAC volume. In NELSON, MVC was non-significantly related to prognosis, with a multivariable-adjusted hazard ratio of 2.74 (95% CI 0.92–4.56) per 500 mm^3^ MVC volume [99].

Adriaans *et al*. [110] followed 338 patients with non-syndromic ascending aortic aneurysms for 6.7 years and found that mean growth rate was 0.2±0.4 mm per year. The optimal interval for imaging surveillance was 3-yearly for aortic size 40–49 mm diameter. Two other studies confirmed this growth rate [111, 112]. Over 5 years, the rate of aortic dissection or rupture was 3882 of 797 692 (0.5%) for patients with initial aortic size of 40–44 mm, 3074 of 183 164 (1.7%) for patients with initial aortic size of 45–49 mm, and 357 of 12 188 (2.9%) for patients with initial aortic size of 50 mm [110]. In a simulation study of 1 million patients, follow-up of patients older than 60–65 years with diameters <50 mm was not cost-effective at a willingness to pay threshold of USD 100 000 per quality-adjusted life-year [113].

#### Statements and health service documents

According to available management documents, aortic calcification does not need reporting or action. The lung cancer screening-based management documents (by ACR and NHS) [5, 6] recommend primary care surveillance/referral in case of a dilated thoracic aorta (of at least 42 or 40 mm, respectively). The former also advises primary care evaluation in case of MPA diameter of at least 31 mm.

#### Summary

TAC is a very frequent finding on CT in lung cancer screening, in contrast to MVC. All prognostic studies show that increasing extent of TAC is related to worse outcomes. Management documents agree on the need to report thoracic aortic dilatation, but currently do not recommend reporting of TAC or severity.

#### Statements

- TAC is a frequent finding in lung cancer screening and is associated with adverse outcomes [90, 99, 109].
- Guidelines do not recommend clinical assessment of TAC.
- Referral of participants with significantly dilated thoracic aorta (≥40 or 42 mm) is recommended in guidelines.
- Recent evidence suggests a better threshold of ≥45 mm and cost-effectiveness of follow-up ≥50 mm.

#### Suggested research

- What real-world benefit may be derived from quantification of TAC?
- Does the detection of TAC lead to changes in patient management, treatment and outcome in the screening context?
- What is the cost-effectiveness of reporting TAC estimations?
- Does the detection of dilated MPA lead to changes in patient management, treatment and outcome in the screening context?

#### Mediastinal lesions

Distinguishing thymic hyperplasia from other mediastinal lesions is important because the former confers no benefit from any medical or surgical intervention [114].

#### Evidence review

For this section a total of 18 full papers were reviewed. Nine were from lung cancer screening studies, six from studies reporting incidental detection outside lung cancer screening and three were review papers.

#### Anterior mediastinal lesions

In the 9263 participants in ELCAP the prevalence was 0.4% [115] and similarly was 0.7% in a Korean lung cancer screening programme [116]. Anterior mediastinal masses may grow despite being sub-centimetre at baseline, warranting long term follow-up or surgical consultation [117]. Although thymic tumours are considered malignant, many anterior lesions are benign so initial follow-up to detect growth is often the preferred management, with further observation or surgery depending on the findings [118].

Benign resections are common in surgical series. Kent *et al.* [119] reported 27.8% “non-therapeutic” thymectomy, and Ackman *et al*. [120] 43.8%, while Yoon *et al.* [116] reported that only 21.6% of the surgical patients had a thymic tumour. A lack of specificity of CT images was cited as the leading cause for these unnecessary treatments. However, Fang *et al.* [118] reported that, by using CT and MRI, 94.5% of the interventions were correct (some of the cysts were eventually operated on because MRI images showed the possibility of a cystic thymoma), but only 76.7% would have been correct if based on CT only.

#### Statements and health service documents

In their white paper, the ACR recommend CT follow-up at 3 months when image findings are inconclusive, but the QRG suggests contrast-enhanced MRI or CT if the mass is soft tissue or mixed density [5, 22]. However, this statement was consensus-based due to a lack of definitive evidence [5, 121]. The NHSE QAS recommends reporting size, position and whether cystic, and recommends clinical review. Options for work-up include positron emission tomography/CT/MRI; cystic lesions do not require further investigations [6].

#### Oesophageal lesions

In 9263 ELCAP participants, only two oesophageal tumours were found at baseline [115]. Guidelines and statements recommend that findings such as significant dilatation, diffuse wall thickening, or focal lesions should be reported because they are clinically significant and may need further radiological or endoscopic studies [5, 122].

#### Mediastinal lymphadenopathy

It has been shown that the prevalence of lymphadenopathy in lung cancer screening CT is between 1% and 6% [2, 123]. In the NLST, most enlarged mediastinal lymph nodes were reactive to an infectious process, pulmonary oedema and diffuse lung disease, and needed no further work-up. Very few cases were related to metastatic lung cancer [124]. In participants with pulmonary nodules no larger than 4 mm, 1.6% had mediastinal or hilar lymphadenopathy [125]. However, participants with enlarged (>10 mm) noncalcified mediastinal lymph nodes at initial CT had a greater chance of being diagnosed with lung cancer than those without enlarged nodes (17.1% *versus* 3.9%; p<0.001). In this secondary unplanned analysis, larger lymph node size was related to higher incidence of lung cancer at a later stage, increased death rate and reduced survival, and suggested more aggressive management. The time to a lung cancer diagnosis was also shorter in the lymphadenopathy group, as most cases were diagnosed within the first 3 years, although the majority were within the first year [126].

#### Statements and health service documents

The ACR recommend that the threshold size is 15 mm on the short axis and that texture or density (if enlarged) should be described [121]. In addition, clinical history and other pulmonary findings are essential in managing enlarged mediastinal lymph nodes *via* a lung cancer or screening review multidisciplinary team [121]. If lymphadenopathy is explained by a benign condition, then observation is recommended but isolated enlarged nodes require investigation [126–128].

#### Summary

Anterior mediastinal lesions are mostly benign, and studies have demonstrated that with careful evaluation, the high benign resection rate seen in some studies can be avoided. Oesophageal pathological findings are usually benign, but may need evaluation if causing symptoms. Lymphadenopathy is infrequent but needs to be explained as it can be associated with a greater chance of lung cancer, although the overall prevalence of cancer remains low.

#### Statements

- Anterior mediastinal masses may be stratified according to their size, position and density/texture [6].
- Higher risk anterior mediastinal masses are best investigated with contrast-enhanced MRI or CT [5, 6].
- Although oesophageal malignancies are uncommon [115], benign pathology may be clinically relevant.
- Mediastinal and hilar lymphadenopathy >15 mm on the short axis that is unexplained may require further investigation and work-up, or at least short interval scanning (3 to 6 months). Morphological assessment of lymph nodes may also be useful [121, 126–128]. A threshold <15 mm will lead to many unnecessary referrals in the context of screening.

#### Suggested research

- What additional predictive factors can help stratify mediastinal masses?
- Is there a role for radiomics or artificial intelligence to further characterise mediastinal masses?

#### Thyroid lesions

Thyroid lesions are readily detected by CT, although ultrasound is better able to characterise them [129].

#### Evidence review

A total of five full papers were reviewed that contained information about thyroid lesions detected in the process of lung cancer screening. European and Canadian CT lung screening trials found that thyroid lesions were reported in less than 5% of patients [69, 70, 130]. A review of 26 722 participants in the NLST found a total of 291 people (1.1%) who had thyroid-related anomalies at baseline (n = 135; 0.5%), 1 year (n = 83; 0.3%) or 2 years (n = 73; 0.3%) [131]. Overall, they found 1.1% general thyroid findings and 0.7% incidental thyroid nodules. In the clinical follow-up of the patients, 35 thyroid cancers were diagnosed in the LDCT arm (25 in the chest radiograph arm of NLST, with no significant difference). The incidence of thyroid cancer was 2.1 per 10 000 person-years of follow-up using LDCT. Interestingly, 16 cases in the LDCT arm had a previously reported abnormality above the diaphragm and, among them, 11 had a specific thyroid abnormality diagnosis in the radiological report. The relative risk of thyroid cancer during follow-up where an abnormality above the diaphragm was identified was 7.8 (95% CI 4–15.1). The incidence rate was 104.6 per 10 000 person-years in those with previously reported thyroid-associated abnormality *versus* 1.4 in non-previously reported thyroid abnormality (relative risk 72.5, 95% CI 37–153). Only three deaths related to thyroid cancer occurred, with a 5-year survival of 88.6% (95% CI 77.1–99.5%). Globally, thyroid cancer accounted for 0.1% of all study-reported deaths.

A retrospective analysis of the LDCT cohort in the NLST, *i.e*. 221 patients aged 55–74 years of age with thyroid incidentalomas performed in 2018, found that a 20 mm rather than a 10 mm threshold achieved a better balance between unnecessary further work-up of benign nodules and delaying diagnosis of thyroid malignancy [132]. A previous review article of incidental findings in lung cancer screening has highlighted the phenomenon of overdiagnosis in the setting of indolent thyroid cancers, observing that malignant thyroid nodules may often represent papillary thyroid cancer, which has close to a 100% 10-year survival rate [132].

#### Statements and health service documents

The ACR QRG recommends referral for thyroid ultrasound and clinical evaluation for nodules ≥15 mm or with suspicious features [5], in line with their more generic advice for patients aged ≥35 years [133]. The NHSE QAS advises reporting only if there is local lymphadenopathy or punctate calcification [6].

#### Summary

Fewer than 5% of CT screening scans detect thyroid abnormalities and because of the eligible age range for screening, most of these are benign. CT is not able to characterise thyroid abnormalities as well as ultrasound can, so guidelines recommend referral of nodules ≥15 mm in size or with suspicious features.

#### Statements

- Thyroid abnormalities are seen on <5% of screening CT scans [69, 70, 130].
- Most of these are benign or indolent [131].
- Guidelines/consensus statements recommend referral for nodules ≥15 mm or those with suspicious features, such as local lymphadenopathy or punctate calcification.
- Evidence has suggested a 20 mm nodule size cut-off for referral may achieve a better balance of avoiding unnecessary work-up of benign nodules [132].

#### Suggested research

- What is the cost and impact on morbidity and mortality in the lung cancer screening population of reporting and investigating thyroid nodules according to guidelines?

### Breast nodules

#### Evidence review

17 full papers were reviewed. Five full papers were relevant to this topic.

#### Prevalence

In a retrospective review of 5201 participants (34% women) aged ≥50 years and screened annually for 5 years in Italy, Rampinelli *et al*. [134] found only one breast cancer, which was diagnosed in the third round. Another lung cancer screening study with a similar design from the Mayo Clinic found three breast cancers and 17 breast nodules out of 210 extrathoracic findings in 1520 participants (48% women) [72]. A retrospective review of CT reports from 4703 (54% women) current or former smokers considered at risk for developing lung cancer who underwent screening found an incidental breast lesion in 37 participants (4%) [130]. Of these, three were breast cancer. 28 out of 37 participants (76%) were referred for further dedicated breast imaging, with most incidental breast findings (81%) detected in the first screening round. A further retrospective review of 320 LDCT-screened participants reported one breast nodule, without further data on characterisation or diagnosis [2]. The large retrospective analysis on incidental findings in 17 309 participants of the NLST does not mention incidental breast abnormalities [135].

#### Statements and health service documents

The NHSE QAS for the TLHC recommends that size and site is reported, and referral made to breast services if the abnormality is not previously known about or if further information is not available. The ACR QRG on incidental findings states that when coarse calcifications or a cyst without associated solid component is detected, no further action is necessary. For all other nodules, masses or asymmetric densities, further work-up with mammography and/or breast ultrasound is advised [5, 6].

#### Summary

Breast cancer detected by screening for lung cancer is uncommon and rates from studies are variable. Given how common breast cancer is, some studies might have been expected to detect breast cancer more often.

#### Statements

- The rate of breast cancer seen in lung cancer screening is variable and very low in some studies [72, 130, 134].
- Most detected breast lesions are benign [72, 130, 134].
- Guidelines recommend referral of any breast lesion that was not previously known, or lesions that are not clearly cystic.

#### Suggested research

- What is the prevalence of breast lesions in screening and what proportion of these are malignant?
- How do breast cancer screening and lung cancer screening interact in terms of breast cancer detection, participation and adherence?

### Adrenal lesions

#### Evidence review

18 full papers were reviewed. Of these, seven full papers were found that were relevant. Hu *et al.* [136] investigated the frequency and progression of enlarged adrenal glands in baseline and annual repeat rounds in 4776 screening participants. They defined an enlarged adrenal gland as ≥6 mm in maximum diameter. Adrenal enlargement was found in 4% (202/4776) of participants, with age and current smoking being significant predictors. During follow-up of 133 participants with adrenal enlargement <40 mm, 48 (36%) enlarged, although none by more than 10 mm. The remaining 85 (64%) showed no growth. None of the 197 participants diagnosed with lung cancer after baseline enrolment had adrenal enlargement. 18 out of the 202 participants with adrenal enlargement (9%) were diagnosed with lung cancer after 9976 person-months of follow-up compared to 179 (4%) participants without adrenal enlargement after 172 388 person-months. In five participants (0.04%) adrenal enlargement was a new finding. Size did not increase beyond 40 mm on follow-up in any participant and any increase was less than 10 mm after 1 year. The authors concluded that 1-year follow-up was adequate.

Rampinelli *et al*. [134] found only one adrenal pheochromocytoma (21 mm lesion, density >10 HU) in 5201 participants. In the sample of 1929 participants in the NELSON trial, adrenal lesions were categorised as “possibly clinically relevant” if their attenuation was >10 HU. Only one participant was identified, and this was a benign cyst; there were 13 lower attenuation lesions [70]. Out of 17 309 participants of NLST, 419 (2.4%) had an adrenal abnormality, of which 207 (1.2%) were categorised as potentially significant [135]. None of the adrenal abnormalities turned out to be a malignancy. Data regarding smaller studies show an incidence of adrenal masses or abnormalities (not further specified) ranging from 2.0% to 3.8% [2, 72, 81].

#### Statements and health service documents

The NHSE QAS for the TLHC recommends reporting size and attenuation. Lesions <10 mm or <10 HU do not require referral. Lesions between 10 and 40 mm and >10 HU also do not require action, but participants should have annual follow-up. Lesions larger than 40 mm should have endocrinology referral. The ACR QRG states that lesions that do not require referral or work-up are: adrenal calcification, nodule <10 HU, soft density nodule <10 mm or adrenal nodule that is stable for ≥1 year. Any other nodule or mass should be referred for work-up with contrast-enhanced CT or MRI [5, 6].

#### Summary

Adrenal abnormalities and adrenal enlargement are common incidental findings. Most findings are benign, and malignancy is reported as very rare. Repeat screening rounds offer adequate follow-up of adrenal abnormalities that are <40 mm in largest diameter and/or have a density >10 HU.

#### Statements

- In the context of lung cancer screening, most incidental adrenal lesions up to 40 mm in size are benign [136].
- Guidelines state that lesions 10–40 mm or with attenuation >10 HU can be followed up at the next annual screening round or referred for further evaluation with contrast-enhanced CT or MRI. Adrenal lesions stable on CT over 12 months also may not require further investigation [5, 6].
- Lesions <10 mm or <10 HU in density do not require further investigation [5, 6, 135].

#### Suggested research

- Does referral of adrenal lesions for contrast-enhanced CT or MRI confer any benefit in the context of lung cancer screening?
- Is there a role for artificial intelligence to safely characterise adrenal lesions and prevent further imaging with contrast-enhanced CT or MRI?

#### Other generic topics

The following topics received a full evidence review but were considered suitable for brief comments agreed by the task force members. For all these topics, reporting was felt to be straightforward and unlikely to vary to any significant extent.

#### Evidence review

In total, 24 full papers were reviewed for these topics and each of the current guidance documents were also reviewed.

#### Tuberculosis

LDCT is known to detect tuberculosis (TB) and, in an at-risk population, reduce the specificity of lung cancer screening by over-diagnosis. TB sequelae were identified in 13% of lung cancer screening participants (OR with one nodule 1.22, 95% CI 1.02–1.45; p = 0.03) in a high prevalence country [137]. If active TB is suspected, indicate a differential diagnosis.

#### Bronchial wall thickening

Xie *et al*. [138] reported bronchial wall thickening in airways in heavy smokers with chronic respiratory symptoms and at high risk of chronic bronchitis in lung cancer screening. Quantitatively assessed CT emphysema, air trapping and bronchial wall thickness each contain independent diagnostic information for COPD in the absence of lung function testing and may influence lung cancer screening strategy [139]. However, the NHSE QAS does not recommend reporting of bronchial wall thickening and the ACR QRG combines bronchial wall thickening with emphysema and suggests that primary care review may be appropriate [5, 6].

#### Respiratory bronchiolitis-associated interstitial lung disease

Sverzellati *et al.* [16] reported a prevalence of respiratory bronchiolitis-associated interstitial lung disease (RB-ILD) of 15.7% in their lung cancer screening cohort. The NHSE QAS suggests reporting RB-ILD, although the only treatment is smoking cessation [6].

#### Bone abnormalities

LDCT is known to detect a large variety of bony abnormalities that are either common and have straightforward reporting and management, or uncommon but have defined management guidelines that can be followed. These include osteoporosis, diffuse idiopathic skeletal hyperostosis, spondylosis, spondylodiscitis and lytic or sclerotic bone lesions. Osteoporosis has been shown to be more severe in smokers in the context of lung cancer screening [140–142]. It may also be a predictor of all-cause mortality in lung cancer screening [143]. Opportunistic screening of osteoporosis might develop in the coming years as automated tools for quantification of bone mineral density and automated fracture detection will be available. These readouts might then become part of the incidental findings section of the lung cancer screening report. The ACR QRG suggests management based on L1 attenuation: if 100–130 HU (osteopenia) consider primary care evaluation, and if <100 HU (osteoporosis) consider dual-energy X-ray absorptiometry in addition. The NHSE QAS suggests secondary referral in case of >50% loss of vertebral height in at least one vertebra [5, 6].

#### Liver lesions

In non-enhanced LDCT, the detection and characterisation of small lesions is difficult. The NLST analysis is the only study large enough to comment on the prevalence of hepatocellular cancer which was 0.05%, a total of eight cancers from 360 hepatobiliary lesions reported [135]. The ACR QRG recommends referring all soft tissue lesions >1 cm for further imaging with contrast and the NHSE QAS refers to detailed management based on the ACR white paper [144]. Table 2 summarises an approach to reporting and management.

**Table 2:** Reference guide for the management of incidental findings on lung cancer screening computed tomography (CT)

Finding	Reporting recommendation	Suggested action if baseline or new
**If clinical information is available (as in some European sites), this should be considered in producing the report and recommendation; however, this table assumes no clinical information is available**		
**Pulmonary**		
Emphysema	Classify as: - mild (<25%) - moderate (25 − 50%) - severe >50%	Refer those with moderate to severe radiological emphysema for clinical assessment Smoking cessation referral for all current smokers
Interstitial lung abnormalities (ILA)	Report all ILA	Surveillance as part of a screening programme, *or* ILA involving more than 5% of either the whole lungs or a lung zone could be referred for specialist review
Bronchiectasis	Report bronchiectasis when moderate or severe (defined as internal bronchial luminal diameter >2 times that of adjacent artery)	Refer for clinical assessment if moderate or severe
Respiratory bronchiolitis-associated interstitial lung disease (RB-ILD)	Report presence of RB-ILD	Smoking cessation referral
Consolidation	Classify as: - likely inflammatory - possibly malignant	Refer to lung cancer service if possibly malignant Repeat CT at 6 weeks or 3 months if likely inflammatory
Pleural effusion/thickening	Report size and laterality	Refer for clinical assessment and work-up if suspicious appearances including a new effusion, malignant appearing pleural thickening or mass
Pleural plaques	Reporting pleural plaques may be appropriate where compensation is offered for their presence^#^ Reporting of pleural plaques may be appropriate is their presence is to be used in lung cancer risk assessment^#^	Ensure that no clinical activity is generated for benign appearances
Tuberculosis (TB)	Report if active TB likely and differential diagnoses	Referral into local TB service
Bronchial wall thickening	Do not report	No action required
**Extrapulmonary**		
Coronary calcification (CAC)	Report CAC Classify (using simple visual scoring) as: - None - Mild - Moderate - Severe	Clinician assessment of cardiovascular risk if moderate or severe CAC present Primary preventive measures (if not already taking)
Aortic valve disease	Report aortic valve calcification (AVC) if moderate or severe Classify using simple visual scoring	Refer those with moderate or severe AVC for evaluation with echocardiography
Thoracic aortic calcification/dilatation	Do not report thoracic aortic calcification Thoracic aorta diameter - <45 mm: do not report - ≥45 mm: report	Referral for further assessment for those with thoracic aorta >45 mm diameter according to local guidelines/pathways
Mediastinal mass	Report size, position and density/texture	Cystic lesions do not require further assessment^#^ Options for management include surveillance as part of the screening programme, *or* work-up with PET/CT/MRI, depending on clinical assessment
Mediastinal lymph nodes	Report mediastinal and hilar lymphadenopathy ≥15 mm short axis	≥15 mm short axis and no explainable cause refer for clinical assessment Surveillance options include short interval CT scan at 3 − 6 months
Thyroid abnormalities	Report nodules ≥15 mm^#^ or those with suspicious features, such as local lymphadenopathy or punctate calcification	Referral for further investigation for nodules ≥20 mm or those with suspicious features
Cardiac decompensation/pericardial effusion	Pericardial fluid - Trivial/small: do not report - Moderate/large: report	Referral for echocardiography/clinical assessment for those with moderate/large pericardial effusions
Oesophageal lesions	Report significant dilatation, diffuse wall thickening or focal lesions	Referral for further assessment according to local guidelines/pathways
Abdominal aortic aneurysm (AAA)	Report all AAA	Referral for further assessment/surveillance according to local guidelines/pathways
Breast nodules	Report size, site, calcification and density	Refer any breast lesion that is not previously known, or lesions that are not clearly cystic, and/or not coarsely calcified, for triple assessment according to local guidelines/pathways
Liver lesions	Report size and attenuation	Benign features; sharp margin and homogenous low attenuation (≤20 HU), (focal) fatty sparing or deposition: do not require further investigation^#^ Lesions <1 cm: no further investigation (unless the patient is high risk cirrhosis or other hepatic risk factors)^#^ Lesions ≥1 cm and no benign features: referral for further investigation with contrast-enhanced CT/ultrasound/MRI
Renal lesions	Report size, site, attenuation and calcification	Homogenous hypodense or hyperdense cysts <3 cm do not require further investigation^#^, larger lesions should be evaluated in the next screening round Soft tissue or mixed density renal mass >1 cm: refer for further assessment with contrast-enhanced CT or MRI
Bone abnormalities	Check attenuation at level of L1 and report if: - 100–130 HU: osteopenia - <100 HU: osteoporosis An alternative approach is to report >50% loss of vertebral height in at least one vertebra	Referral for risk assessment (±DEXA) and bone protection for ≤130 HU or >50% loss of vertebral height
Adrenal lesions	Report size and attenuation	Lesions <10 mm or <10 HU density do not require further investigation^#^ Lesions 10–40 mm or with attenuation >10 HU can be followed up at the next annual screening round *or* referred for further evaluation with contrast-enhanced CT or MRI Adrenal lesions stable on CT over 12 months may not require further investigation

# : where there is no action required, it may not be required to report the finding at all; this is at the discretion of individual programmes. PET: positron emission tomography; MRI: magnetic resonance imaging; DEXA: dual-energy X-ray absorptiometry.

#### Renal lesions

As for liver lesions, detection and characterisation of small lesions is difficult. In the NLST study, the prevalence of renal cancer was 0.08%: 14 cancers from a total of 420 lesions reported. Recommendations from guidance documents draw on the detailed recommendations in the ACR white paper for incidental renal lesions [145]. Table 2 summarises an approach to reporting and management.

#### Gall bladder

LDCT is known to frequently detect gall stones without complications, which would not be reported in the screening context, unless obvious complications or masses are observed [146].

#### Cardiac decompensation/pericardial disease

No evidence was found for reporting of cardiac decompensation during lung cancer screening. The ACR QRG considers pericardial fluid and suggests that those with moderate or large pericardial effusions should be referred to primary care providers, but trivial or small amounts of fluid need not be reported [5].

#### Skin abnormalities

No evidence was found for detection of skin lesions during lung cancer screening.

#### Previous surgery

LDCT is known to detect a certain number of surgical procedures previously performed in screening candidates. Reporting of those previously performed surgical procedures is considered as straightforward without any expected variation. As previous clinical imaging is not available to screening programmes, detailed analysis of previous surgery is generally not advised.

#### Abdominal aortic aneurysm

Management guidelines for aortic aneurysm are well defined and these should be followed.

## CONCLUSION

The management of incidental findings detected during lung cancer screening is likely to be a key factor in overall effectiveness and cost-effectiveness of the programme. Although individual countries and programmes may differ slightly in their approach to management, it is important that evidence-based practice is employed. It is acknowledged that trials have employed different imaging protocols, many of which are now superseded so that the prevalence of incidental findings may differ. Further research is suggested for some of the topics, but in general, it is important to include research into radiomics and artificial intelligence. However, the comprehensive systematic review of the evidence has enabled this expert collaborative group to provide statements on how best to manage incidental findings to minimise harm and maintain the opportunity for benefit.

This document was endorsed by the ERS Executive Committee on 17 July 2023, by ESTS on 31 July 2023, by ESTRO on 31 July 2023, by the ESR Board of Directors on 13 July 2023, by ESTI on 28 July 2023, and by EFOMP on 28 July 2023.

## Supplementary Material

ezad302_Supplementary_Data

## SUPPLEMENTARY MATERIAL

**Funding:** This work was supported by the European Respiratory Society (grant: number TF-2019-14; MILCa). Funding information for this article has been deposited with the Crossref Funder Registry.

**Conflict of interest: **All declarations are outside the submitted work. Unless otherwise stated, authors do not make any relevant disclosures. A. Brunelli declares honoraria from AstraZeneca, Ethicon, Medtronic, MSD and Roche. J. Chorostowska declares honoraria from AstraZeneca, MSD, Pfizer, Takeda, Amgen, Grifols, CSL Behring, Novartis, Chiesi, Celon Pharma, Adamed and Mero Biopharma. A. Devaraj declares consulting fees from Boehringer Ingelheim, Roche, Vicore and Brainomix, and personal stock/options in Brainomix. D. Deruysscher declares grants/honoraria from AstraZeneca, BMS, Beigne and Philips. H. Prosch declares grants and honoraria from Boehringer Ingelheim, AstraZeneca, Siemens and MSD. A. Kerpel-Fronius declares honoraria from MSD and Boehringer Ingelheim, and conference expenses from Roche and Bracco. W. van Geffen is local PI for trials funded by MSD and Roche. H-U. Kauczor declares honoraria and grants from Boehringer Ingelheim, Philips, Siemens and MSD, and honoraria from Sanofi. P.M. Putora declares grants to his institution from AstraZeneca and Bayer. M-P. Revel declares honoraria from MSD, Boehringer Ingelheim, GE Healthcare, Bracco and Gleamer, grants from the French Ministry of Health and French Cancer Institute, and software from Aidence, Mevis, Coreline and Gleamer. R. Vliegenthart declares honoraria from Siemens Healthineers and Bayer, and research grants from the Dutch Cancer Foundation, Siemens Heathineers, the Dutch Heart Foundation and the Netherlands Organisation for Scientific Research. A. Snoeckx declares grants from the Foundation Agaist Cancer and the Flemish Cancer Society, and advisory board work for Agfa. G. Veronesi declares honoraria from AstraZeneca, Medtronic and Roche, and grants from INAIL and IARC. D.R. Baldwin declares honoraria for speaking from Roche, MSD and AstraZeneca, and research grants from CRUK, NIHR, Innovate UK, Small Business Research Initiative, NHS Digital, NHS England, Roy Castle Lung Foundation, European Commission Horizon and Yourshire Cancer; and is advisor to the UK National Screening Committee.
