# Supplementary material for: ERS/ESTS/ESTRO/ESR/ESTI/EFOMP statement on management of incidental findings from low dose CT screening for lung cancer
Source: Eur J Cardiothorac Surg. 2023 Oct 7;64(4):ezad302. doi: 10.1093/ejcts/ezad302 (PMC10876118; doi:10.1093/ejcts/ezad302)
Supplement: ezad302_Supplementary_Data [file ezad302_supplementary_data.pdf]

ERS/ESTS/ESTRO/ESR/ESTI/EFOMP Statement on Management of Incidental Findings from Low-Dose CT screening for Lung Cancer (MILCa)

Appendices

Appendix A: Search Terms

MILCa search strategy

SCOPE:

Narrative review on pre-defined essential incidental findings in LDCT lung cancer screening

SEARCH STRATEGIES:

Platforms used to perform searches were Medline via PubMed and EMBASE

1. **Broad search on LDCT lung cancer screening = 1663 abstracts without duplicates (25/11/2021)**
2. **Narrowed search on LDCT lung cancer screening AND incidental finding = 56 abstracts (25/11/2021)**
3. **NOT defined yet but probably needed: separate searches for CT (Thorax) AND single pre-defined essential incidental findings**

1. Broad search on LDCT lung cancer screening

(exp mass screening/ or exp early diagnosis/ or screening.tw or early diagnos*.tw) and (lung neoplasms/ or bronchial neoplasms/ or carcinoma, bronchogenic/ or carcinoma, non-small-cell lung/ or small cell lung carcinoma/ or pancoast syndrome/ or lung neoplasm*.tw or lung cancer*.tw or lung carcinoma*.tw or lung tumour*.tw or lung tumor*.tw or pulmonary neoplasm*.tw or pulmonary cancer*.tw or pulmonary carcinoma*.tw or pulmonary tumour*.tw or pulmonary tumor*.tw or bronchial neoplasm*.tw or bronchial cancer*.tw or bronchial carcinoma*.tw or bronchial tumour*.tw or bronchial tumor*.tw or bronchogenic neoplasm*.tw or bronchogenic cancer*.tw or bronchogenic carcinoma*.tw or bronchogenic tumour*.tw or bronchogenic tumor*.tw or pancoast* syndrome*.tw or pancoast* tumor*.tw or pancoast* tumour*.tw) and (exp Tomography, X-Ray/ or Tomography Scanners, X-Ray Computed/ or CT*.tw or Scan*.tw or Tomograph*.tw or Tomodensitometr*.tw) and (smokers/ or exp smoking/ or tobacco/ or exp "Tobacco Use"/ or exp tobacco products/ or smoker*.tw or tobacco smok*.tw or tobacco consumption.tw or cigaret*.tw or high risk*.tw) = 1668– 1663 without duplicates (25/11/2021)

AND (Incidental Findings/ or incidental finding*.tw OR incidental discover*.tw) = 56 (25/11/2021)

__2. Narrowed search on LDCT lung cancer screening AND incidental finding__

Search strategy 1 AND (Incidental Findings/ or incidental finding*.tw OR incidental discover*.tw) = 56 (25/11/2021)

Appendix B: PRISMA

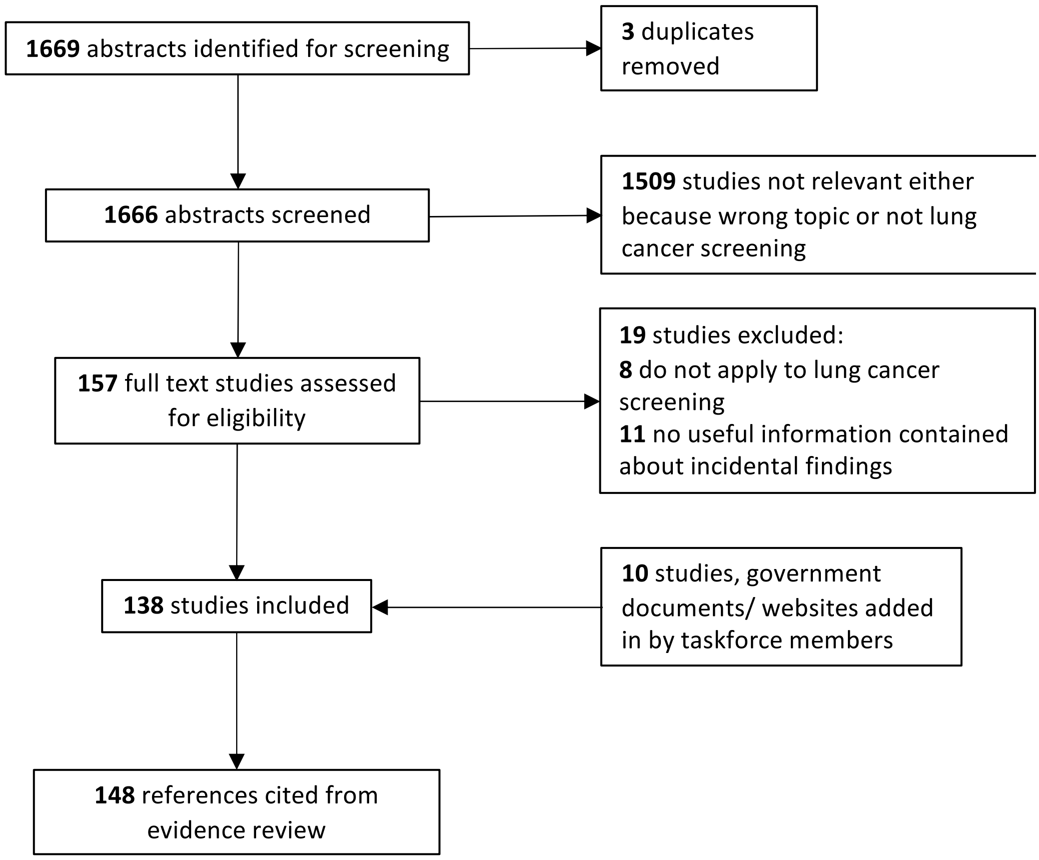

APPENDIX C

Summary of research questions in the management of incidental findings suggested on LDCT

Interstitial lung abnormality

- How should ILA be quantified?
- What is the role of artificial intelligence in the classification and quantification of ILA?
- How should ILA and ILD be consistently differentiated?
- How should you follow-up ILA and does it affect prognosis?
- Is the identification of ILA an opportunity for therapeutic prevention of progression, and does this differ according to sub-type of ILA on CT?
- What are the thresholds for further action (i.e. clinical assessment) beyond observation along with the routine screening intervals?

Emphysema

- What are the benefits of screening patients with emphysema (as a comorbidity) given the increased lung cancer mortality and all-cause mortality in this cohort?
- Given the relationship between emphysema and lung cancer risk, should emphysema (severity) on screening LDCT influence subsequent LDCT screening and its interval?
- Does the detection of emphysema and subsequent investigation of an individual for COPD lead to changes in patient management, behaviour (such as smoking cessation), treatment (including pharmacotherapy) and most importantly outcome in the screening context?
- What is the cost effectiveness of COPD case finding through reporting emphysema in the context of screening for lung cancer?
- How can the method for quantification of emphysema in LDCT be optimized, and is there additional value of emphysema quantification?

Bronchiectasis

- How should bronchiectasis severity and extent be quantified?
- What is the role of artificial intelligence in the classification and quantification of bronchiectasis?
- Does the detection and quantification of bronchiectasis during screening result in meaningful beneficial changes to patient management, treatment (including pharmacotherapy) or patient outcome?
- What is the cost effectiveness of detecting bronchiectasis in a lung cancer screening programme?

Consolidation

- What are the outcomes of protocols that manage screen-detected consolidation?
- Can artificial intelligence be used to better categorize consolidation at a single timepoint CT?

Coronary artery calcification

- What is the impact of CAC scoring provoked primary prevention on long term outcomes?

- How does risk stratification with CAC supplement existing risk stratification tools such as Q-Risk in individuals with a strong smoking history?
- Are quantitative methods of CAC evaluation better at predicting and improving outcomes?
- How far does appearance of (severe) CAC change the overall risk stratification including eligibility for the screening program?
- What is the role of artificial intelligence in the quantification of CAC and what is its impact on accuracy of prevention?
- What is the cost effectiveness of reporting CAC?
- What is the impact on prognosis and management of the absence of coronary calcium on LDCT in lung screening populations?

Aortic valve calcification (AVC)

- How does reporting of moderate/severe AVC in lung cancer screening impact on outcomes?
- What is the cost effectiveness of reporting AVC?
- Do quantitative measurements improve identification of AVC that can be effectively treated, and does quantification lead to better outcomes?
- Can AI be used to quantify AVC?

Other cardiovascular findings

- What real-world benefit may be derived from quantification of thoracic aortic calcification (TAC)?
- Does the detection of TAC lead to changes in patient management, treatment and outcome in the screening context?
- What is the cost-effectiveness of reporting TAC estimations?
- Does the detection of dilated main pulmonary artery lead to changes in patient management, treatment and outcome in the screening context?

Mediastinal masses

- What additional predictive factors can help stratify mediastinal masses?
- Is there a role for radiomics or AI to further characterize mediastinal masses?

Thyroid abnormalities

- What is the cost and impact on morbidity and mortality in the lung cancer screening population of reporting and investigating thyroid nodules according to guidelines?

Breast lesions

- What is the prevalence of breast lesions in screening and what proportion of these are malignant
- How do breast cancer screening and lung cancer screening interact in terms of breast cancer detection, participation and adherence?

Adrenal lesions

- Does referral of adrenal lesions for CE CT or MRI confer any benefit in the context of lung cancer screening?
- Is there a role for artificial intelligence to safely characterise adrenal lesions and prevent further imaging with CE CT or MRI?
